# Traffic-related air pollution associated with prevalence of asthma and COPD/chronic bronchitis. A cross-sectional study in Southern Sweden

**DOI:** 10.1186/1476-072X-8-2

**Published:** 2009-01-20

**Authors:** Anna Lindgren, Emilie Stroh, Peter Montnémery, Ulf Nihlén, Kristina Jakobsson, Anna Axmon

**Affiliations:** 1 Department of Occupational and Environmental Medicine, Lund University, Lund, Sweden; 2 Department of Community Medicine, Lund University, Lund, Sweden; 3 Astra Zeneca R&D, Lund, Sweden; 4 Department of Respiratory Medicine and Allergology, Lund University, Lund, Sweden

## Abstract

**Background:**

There is growing evidence that air pollution from traffic has adverse long-term effects on chronic respiratory disease in children, but there are few studies and more inconclusive results in adults. We examined associations between residential traffic and asthma and COPD in adults in southern Sweden. A postal questionnaire in 2000 (n = 9319, 18–77 years) provided disease status, and self-reported exposure to traffic. A Geographical Information System (GIS) was used to link geocoded residential addresses to a Swedish road database and an emission database for NOx.

**Results:**

Living within 100 m of a road with >10 cars/minute (compared with having no heavy road within this distance) was associated with prevalence of asthma diagnosis (OR = 1.40, 95% CI = 1.04–1.89), and COPD diagnosis (OR = 1.64, 95%CI = 1.11–2.4), as well as asthma and chronic bronchitis symptoms. Self-reported traffic exposure was associated with asthma diagnosis and COPD diagnosis, and with asthma symptoms. Annual average NOx was associated with COPD diagnosis and symptoms of asthma and chronic bronchitis.

**Conclusion:**

Living close to traffic was associated with prevalence of asthma diagnosis, COPD diagnosis, and symptoms of asthma and bronchitis. This indicates that traffic-related air pollution has both long-term and short-term effects on chronic respiratory disease in adults, even in a region with overall low levels of air pollution.

## Background

Traffic-related air pollution is well known to have short-term effects on chronic respiratory disease, exacerbating symptoms and increasing hospital admissions for respiratory causes [1]. Strong effects on symptoms have also been observed in areas with relatively low background pollution [2]. Long-term effects have been disputed, but there is growing evidence that traffic-related air pollution is related, at least among children, to asthma incidence [3-7], decreased lung function development [8,9], and incidence of bronchitic symptoms [4,10].

In adults, studies of long-term effects from traffic-related air pollution have been few, and recent studies have found both positive [11-15] and negative [16-18] associations with asthma, as well as positive [16,19,20] and negative [13,14] associations with COPD. Overall, chronic respiratory disease in adults is heterogenous and involves major exposures, such as personal smoking and occupational exposure, which do not directly affect children. This larger variety of risk factors may complicate research and contribute to inconclusive results in adults.

Self-reported living close to traffic has been associated with prevalence of asthma, but not COPD, among adults in southern Sweden [14]. However, self-reports could be severely biased if people are more aware of (and hence over-report) exposures that are known to be potentially connected to disease, and may not be trustworthy if used as the only exposure estimate [21].

One way of obtaining objective exposure estimates is the use of Geographical Information Systems (GIS) to combine geocoded population data with external traffic exposure data, such as road networks and modeled or monitored traffic pollutants. Since the concentrations of many traffic pollutants decline to background levels within 30–200 m of a road, the level of spatial aggregation may be just as important as the type of proxy when estimating exposure [22,23]. Some studies have found that adverse effects on respiratory disease are best captured with simple variables of traffic density and proximity to roads [24], rather than more complex models of specific pollutants, which are difficult to model with a high resolution. However, air pollutant models do have a number of advantages; for example, they can account for total traffic density, and can also be validated against real measurements, providing more specific estimates of the level of pollution at which adverse effects from traffic can be seen.

In the present study, we made use of a high quality GIS-modeled pollutant database for nitrogen oxides (NO_x _and NO_2_) which has been developed and validated for southern Sweden [25]. The high spatial variability of NO_x _(NO+NO_2_), with traffic as the dominating source, makes it a better proxy for exposure to traffic at the local level, compared with pollutants such as PM_2.5 _which have a more geographically homogenous spread [26]. We also used GIS-based road data and self-reported living close to heavy traffic as proxies for exposure.

### Study aim

The aim of this study was to investigate the association between traffic-related air pollution and asthma and COPD in adults. The outcomes investigated were prevalence of; 1) asthma diagnosis 2) COPD diagnosis 3) asthma symptoms last 12 months, and 4) chronic bronchitis symptoms, in relation to residential traffic exposure.

## Methods

### Study area

**Figure 1:**
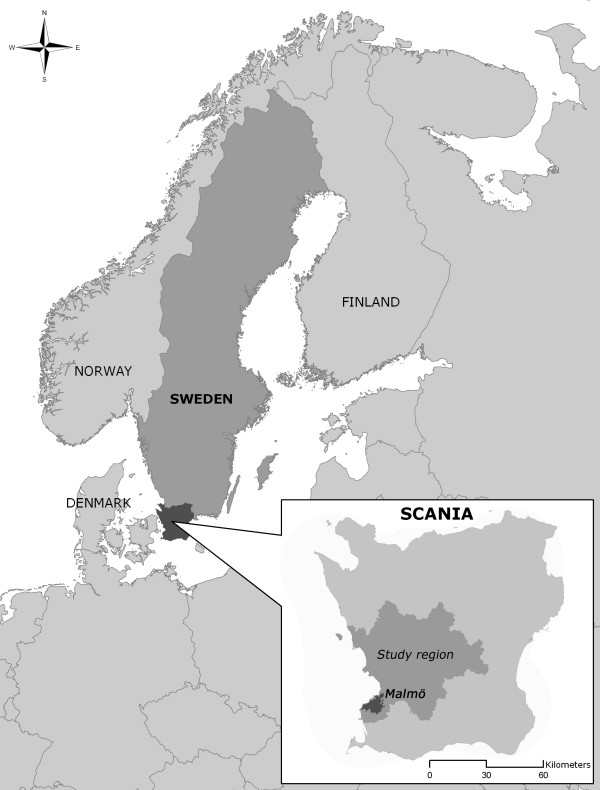
**Study area**. Malmö is the largest city in the study region, which comprises the southwestern part of Sweden.

The study area was the most southwestern part of Sweden (figure 1), the most populated part of the county of Scania. The study area contains 840 000 of Sweden's total population of 8.9 million, and has a population density of 170 inhabitants per km^2 ^(data from 2000). The majority of the population live in six of the communities, the largest of which is Malmö, the third largest city in Sweden, with a population of 260 000. A detailed regional description has previously been given [27]. In the geographical stratification of the present study, "Malmö" refers strictly to the city boundaries of Malmö, not the larger municipality.

The climate in the region is homogenous. Although pollutant levels in the region are low in an European context, they are higher than in the remainder of Sweden [28], due to long-range transport of pollutants from the continent and extensive harbor and ferry traffic.

### Study population & questionnaire

In 2000, a questionnaire was sent to a total of 11933 individuals aged 18–77, of whom 9319 (78%) answered. The study population originated from two different study populations, with 5039 (response rate: 71%) from a new random selection, and 4280 (response rate: 87%) constituting a follow-up group from an earlier selection [29].

The questionnaire dealt with respiratory symptoms, potential confounders such as smoking habits and occupation, and exposures such as living close to a road with heavy traffic [29]. An external exposure assessment was also obtained by geocoding the residential addresses (as of 2000) of both respondents and non-respondents. This was achieved by linking each individual's unique 10-digit personal identity codes to a registry containing the geographical coordinates of all residential addresses.

Non-respondents had a higher mean of NO_x _than respondents; 14.7 μg/m^3 ^versus 13.5 μg/m^3^. To a large extent this was due to a lower response rate in the more polluted city of Malmö (73% vs. 80% in the remaining region).

### Outcome measures

The following outcomes were investigated, as obtained by the postal questionnaires:

• *Diagnosis of asthma*. "Have you been diagnosed by a doctor as having asthma?"

• *Diagnosis of COPD/CBE (Chronic Bronchitis Emphysema)*. "Have you been diagnosed by a doctor as having chronic bronchitis, emphysema, or COPD?"

• *Asthma symptoms during the last 12 months*. "Have you had asthma symptoms during the last 12 months, i.e. intermittent breathlessness or attacks of breathlessness? The symptoms may exist with or without cough or wheezing."

• *Chronic bronchitis symptoms*. "Have you had periods of at least three months where you brought up phlegm when coughing on most days?", and if so, "Have you had such periods during at least two successive years?"

The questionnaire has been published previously [29]. No information regarding year of disease onset was available.

### Exposure assessment

**Figure 2:**
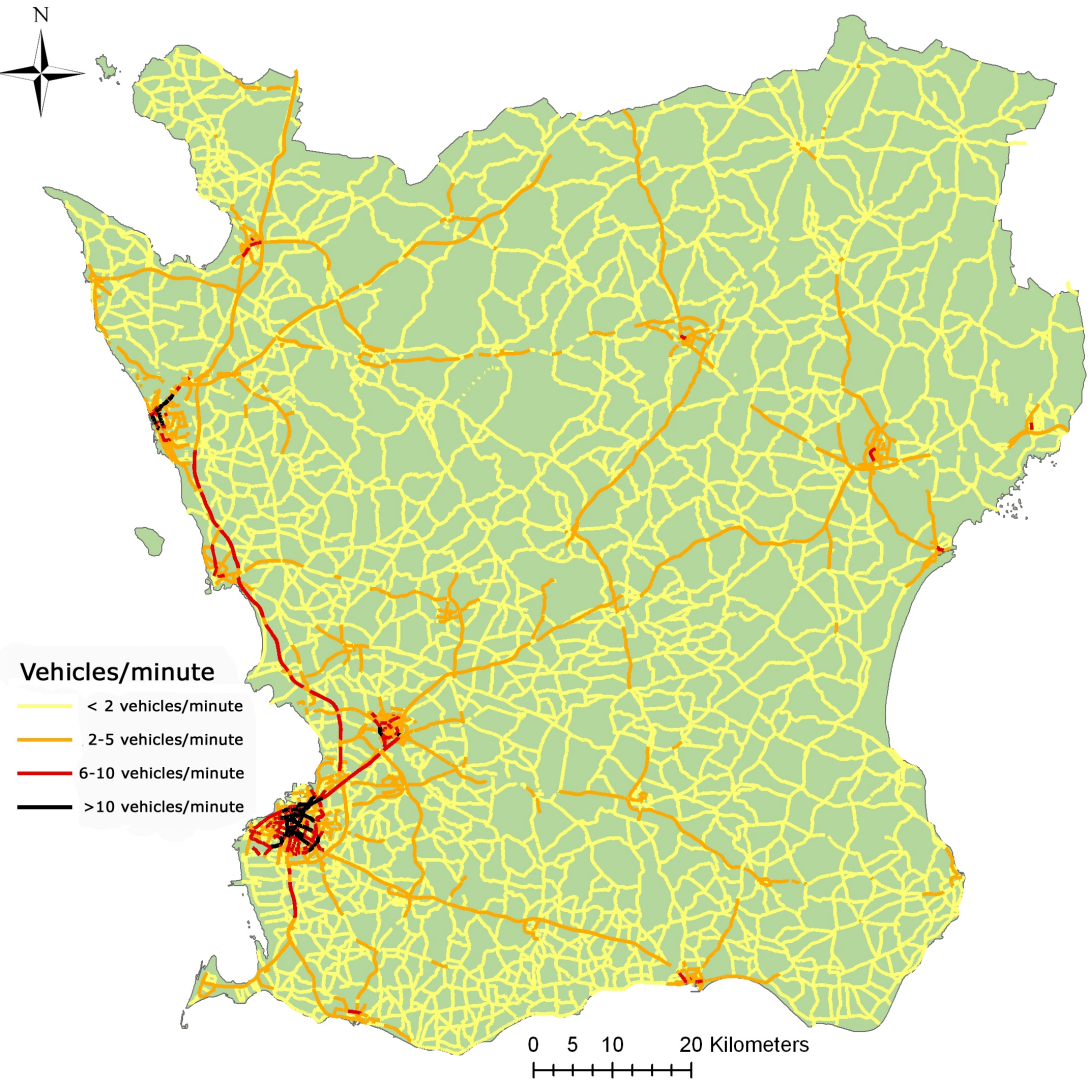
**Regional road network**. Data from the Swedish National Road Network. No heavy road means that no registered road was available in the database, but local traffic could exist. The traffic intensity categories of (0–1, 2–5, 6–10, >10) cars/min corresponds to daily mean traffic counts of (0–2880, 2880–8640, 8640–14400, >14400) cars/day.

**Figure 3:**
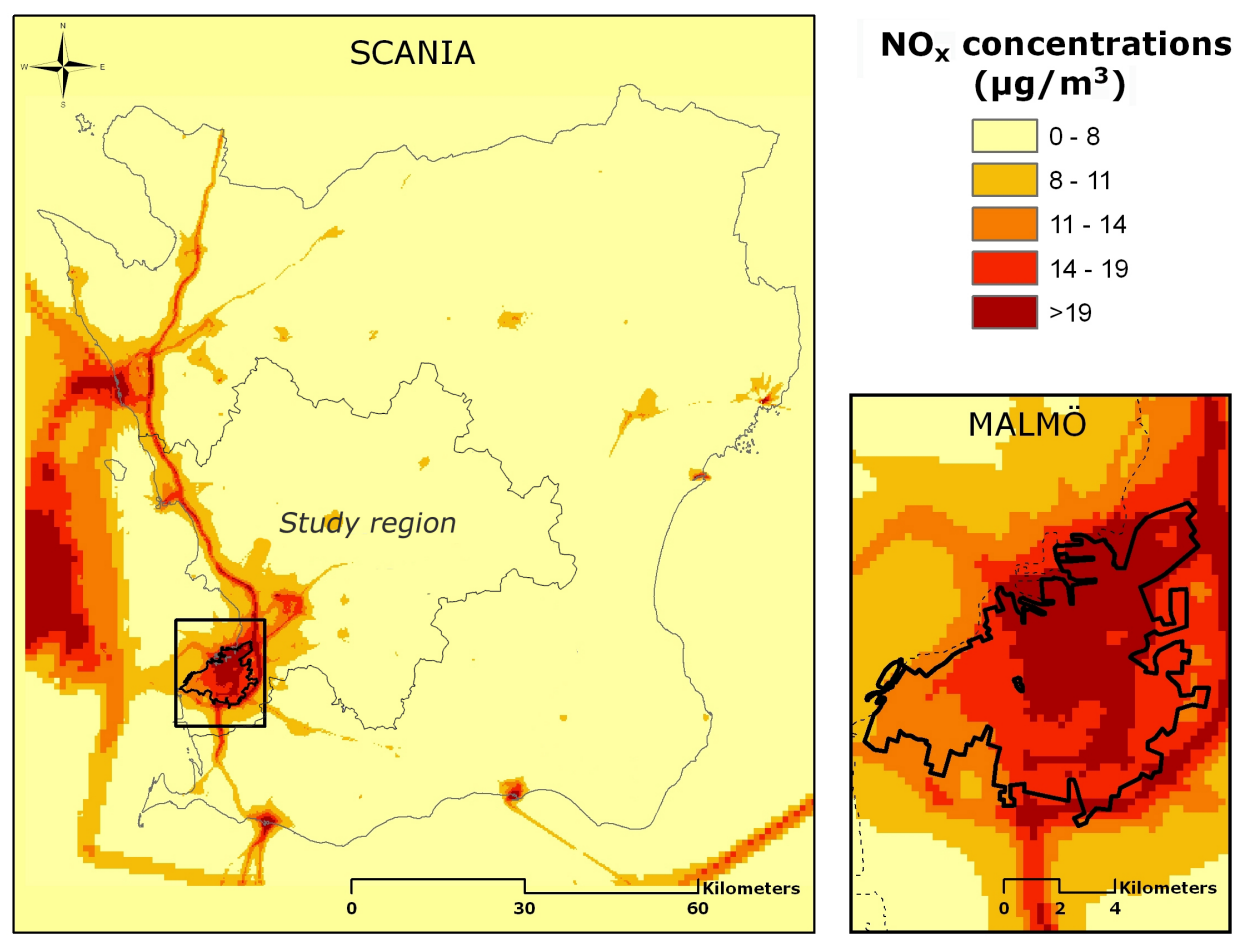
**Modeled levels of NO_x _Dispersion modeled annual average of NO_x_, modeled with a resolution of 250 × 250 m**.

Exposure to traffic-related air pollution was assessed at each participant's residential address in 2000, using three different proxies:

1. Self-reported exposure to traffic. This was obtained from the survey. Exposure was defined as a positive answer to the question "*Do you live close to a road with heavy traffic?*"

2. Traffic intensity on the heaviest road within 100 m. GIS-based registers from *The Swedish National Road Database *[30] provided information about traffic intensity for all major roads in the county (figure 2). To assess exposure to traffic, we identified the road with the heaviest traffic intensity within 100 m of the residence. Traffic intensity was categorized as 0–1 cars/min, 2–5 cars/min, 6–10 cars/min, and >10 cars/min, based upon 24-hour mean levels.

3. Modeled exposure to NO_x _(figure 3). Annual mean concentrations of NO_x _were acquired from a pollutant database, based on the year 2001 [25,31]. Emission sources included in the model were: road traffic, shipping, aviation, railroad, industries and larger energy and heat producers, small scale heating, working machines, working vehicles, and working tools. Meteorological data were also included. A modified Gaussian dispersion model (AERMOD) was used for dispersion calculations; a flat two-dimensional model which did not adjust for effects of street canyons or other terrain, but which did take the height of the emission sources into consideration. Concentrations of NO_x _were modeled as annual means on a grid with a spatial resolution of 250 × 250 m. Bilinear interpolation was used to adjust individual exposure with weighted values of neighboring area concentrations. Concentrations modeled with this spatial resolution have been validated and found to have a high correlation with measured values in the region [25,31].

### Statistics

A categorical classification of NO_x _was used in order to allow analysis of non-linear associations with outcomes. To determine the category limits, the subjects (n = 9274) were divided into NO_x_-quintiles. The five exposure groups used were 0–8 μg/m^3^, 8–11 μg/m^3^, 11–14 μg/m^3^, 14–19 μg/m^3^, and >19 μg/m^3^.

For all measures of exposure, subgroup analyses were made for Malmö and the remaining region. Relative risk was not estimated in exposure groups with fewer than 50 individuals. As few individuals in Malmö had a low exposure to NO_x_, the middle exposure group was used as the reference category for NO_x_, in Malmö. Because of this, NO_x _was also used as a continuous variable for trend analysis using logistic regression. A p-value < 0.05 was regarded as evidence of a trend. Stratified analyses were performed for sex, age, smoking, geographical region (Malmö vs. remaining region), and study population (new random selection vs. follow-up group). Sensitivity analyses of the associations with traffic were also performed by restricting the groups to those with asthma but not COPD, and COPD but not asthma, to exclude confounding by comorbidity. This process was also followed for symptoms.

Relative risk was estimated using Odds Ratios (ORs) with 95% Confidence Intervals (CI). Odds Ratios and tests of trends were obtained by binary logistic regression, using version 13.0 of SPSS.

Sex, age (seven categories), and smoking (smokers/ex-smokers vs. non-smokers) are known risk factors for asthma, and were therefore adjusted for in the model. Socio-Economic Indices (SEI codes, based on occupational status [32]) and occupational exposure (ALOHA JEM [33]) were tested as confounders, using the "change-in-estimate" method [34], where a change in the OR of 10% would have motivated an inclusion in the model.

Neither occupational exposure nor Socio-Economic Indices fulfilled the predetermined confounder criteria, or had any noticeable impact on the risk estimates, and were thus not included in the model.

## Results

A description of the study population in terms sex, age, and smoking, along with the associations with the outcomes, is presented in table 1.

**Table 1:** Description of study population. Disease prevalence in relation to sex, age, and smoking.

		n	Diagnosed asthma	Asthma symptoms	Diagnosed COPD	Chronic b. symptoms
Sex	Men	4341	258(5.9%)	429(9.9%)	172(4.0%)	308(7.1%)
	Women	4975	428(8.6%)	686(13.8%)	243(4.9%)	327(6.6%)
						
Ever smoker	No	4306	291(6.8%)	431(10.0%)	118(2.7%)	217(5.0%)
	Yes	5010	395(7.9%)	684(13.7%)	297(5.9%)	418(8.3%)
						
Age	18–19	135	15(11.1%)	23(17%)	3(2.2%)	9(6.7%)
	20–29	1062	110(10.4%)	141(13.3%)	19(1.8%)	41(3.9%)
	30–39	2045	158(7.7%)	246(12.0%)	61(3.0%)	108(5.3%)
	40–49	1887	112(5.9%)	217(11.5%)	69(3.7%)	101(5.4%)
	50–59	2123	142(6.7%)	237(11.2%)	106(5.0%)	185(8.7%)
	60–69	1586	113(7.1%)	178(11.2%)	115(7.3%)	139(8.8%)
	70–77	478	36(7.5%)	73(15.3%)	42(8.8%)	52(10.9%)

### Association with self-reported living close to traffic

Asthma diagnosis and asthma symptoms in the last 12 months were associated with self-reported traffic exposure (table 2). These results were consistent in a geographical stratification (tables 3, 4).

**Table 2:** Asthma diagnosis and asthma symptoms in relation to traffic.

		Asthma Diagnosis			Asthma Symptoms		
		n	n (%)	OR ^a^	n	n (%)	OR ^a^,
Heavy traffic	No	6041	400(6.6%)	1.00	6041	668(11.1%)	1.00
	Yes	3275	286(8.7%)	1.28(1.09–1.50)	3275	447(13.6%)	1.22(1.07–1.39)
							
Heaviest road within <100 m	no heavy road	3755	269(7.2%)	1.00	3755	419(11.2%)	1.00
	<2 cars/min	2235	149(6.7%)	0.92(0.75–1.13)	2235	263(11.8%)	1.05(0.89–1.24)
	2–5 cars/min	1820	134(7.4%)	1.00(0.81–1.25)	1820	216(11.9%)	1.06(0.89–1.26)
	6–10 cars/min	886	69(7.8%)	1.05(0.79–1.38)	886	126(14.2%)	1.25(1.01–1.55)
	>10 cars/min	578	61(10.6%)	1.40(1.04–1.89)	578	85(14.7%)	1.29(1.00–1.67)
							
NO_x _(μg/m^3^)	0–8	1855	140(7.5%)	1.00	1855	217(11.7%)	1.00
	8–11	1855	146(7.9%)	1.04(0.82–1.32)	1855	213(11.5%)	0.97(0.80–1.19)
	11–14	1855	124(6.7%)	0.85(0.66–1.09)	1855	208(11.2%)	0.94(0.77–1.15)
	14–19	1858	115(6.2%)	0.77(0.60–1.00)	1858	206(11.1%)	0.90(0.74–1.11)
	>19	1851	157(8.5%)	1.05(0.83–1.34)	1851	265(14.3%)	1.21(0.99–1.46)
			p-trend	0.84		p-trend	0.026

^a ^Adjusted for age, sex, and smoking. [OR(95%CI)].

**Table 3:** Geographical stratification. Asthma diagnosis in the city of Malmö and the area outside.

		Malmö			Region outside Malmö		
		n	Asthma diagnosis	OR ^a^	n	Asthma diagnosis	OR ^a^
Heavy traffic	No	1767	109(6.2%)	1.00	4178	283(6.8%)	1.00
	Yes	1877	161(8.6%)	1.35(1.05–1.75)	1343	119(8.9%)	1.28(1.02–1.61)
							
Heaviest road within <100 m	no heavy road	586	40(6.8%)	1.00	3124	224(7.2%)	1.00
	<2 cars/min	1021	66(6.5%)	0.95(0.63–1.43)	1193	82(6.9%)	0.95(0.73–1.23)
	2–5 cars/min	837	57(6.8%)	0.99(0.65–1.51)	961	75(7.8%)	1.07(0.81–1.40)
	6–10 cars/min	663	50(7.5%)	1.12(0.72–1.72)	212	19(9.0%)	1.21(0.74–1.99)
	>10 cars/min	537	57(10.6%)	1.50(0.98–2.31)	31	2	-
							
NO_x _(μg/m^3^)	0–8	13	1	-	1824	138(7.6%)	1.00
	8–11	46	5	-	1792	138(7.7%)	1.01(0.79–1.30)
	11–14	562	39(6.9%)	1.00	1268	83(6.5%)	0.81(0.61–1.08)
	14–19	1325	76(5.7%)	0.79(0.53–1.18)	510	37(7.3%)	0.93(0.64–1.36)
	>19	1698	149(8.8%)	1.18(0.81–1.71)	127	6(4.7%)	0.58(0.25–1.34)
							
			p-trend	0.044		p-trend	0.079

^a ^Adjusted for age, sex, and smoking. [OR(95%CI)].

**Table 4:** Geographical stratification. Asthma symptoms in the city of Malmö and the region outside.

		Malmö			Region outside Malmö		
		n	Asthma symptoms	OR ^a^	n	Asthma symptoms	OR ^a^
Heavy traffic	No	1767	209(11.8%)	1.00	4178	449(10.7%)	1.00
	Yes	1877	263(14.0%)	1.17(0.96–1.43)	1343	178(13.3%)	1.23(1.02–1.49)
							
Heaviest road within <100 m	No heavy road	586	74(12.6%)	1.00	3124	342(10.9%)	1.00
	<2 cars/min	1021	119(11.7%)	0.93(0.68–1.26)	1193	142(11.9%)	1.09(0.88–1.34)
	2–5 cars/min	837	101(12.1%)	0.97(0.70–1.33)	961	112(11.7%)	1.06(0.84–1.33)
	6–10 cars/min	663	97(14.6%)	1.17(0.85–1.63)	212	29(13.7%)	1.24(0.82–1.87)
	>10 cars/min	537	81(15.1%)	1.19(0.84–1.68)	31	2	-
							
NO_x _(μg/m^3^)	0–8	13	1	-	1824	215(11.8%)	1.00
	8–11	46	6	-	1792	205(11.4%)	0.96(0.79–1.18)
	11–14	562	65(11.6%)	1.00	1268	142(11.2%)	0.93(0.74–1.16)
	14–19	1325	146(11.0%)	0.90(0.66–1.23)	510	57(11.2%)	0.95(0.69–1.29)
	>19	1698	254(15.0%)	1.28(0.95–1.72)	127	8(6.3%)	0.50(0.24–1.04)
							
			p-trend	0.002		p-trend	0.344

^a ^Adjusted for age, sex, and smoking. [OR (95%CI)].

COPD diagnosis was associated with self-reported traffic exposure, both for the whole region (table 5) and when geographically stratified (table 6). Chronic bronchitis symptoms were not associated with self-reported traffic exposure (tables 5, 7).

**Table 5:** COPD diagnosis and chronic bronchitis symptoms in relation to traffic.

		COPD Diagnosis			Chronic bronchitis symptoms		
		n	n, (%)	OR ^a^	n	n, (%)	OR ^a^
Heavy traffic	No	6041	243(4.0%)	1.00	6041	401(6.6%)	1.00
	Yes	3275	172(5.3%)	1.36(1.10–1.67)	3275	234(7.1%)	1.11(0.94–1.31)
							
Heaviest road within <100 m	no heavy road	3755	153(4.1%)	1.00	3755	222(5.9%)	1.00
	<2 cars/min	2235	95(4.3%)	1.04(0.80–1.35)	2235	159(7.1%)	1.21(0.98–1.50)
	2–5 cars/min	1820	71(3.9%)	0.96(0.72–1.28)	1820	137(7.5%)	1.30(1.04–1.62)
	6–10 cars/min	886	60(6.8%)	1.57(1.15–2.14)	886	67(7.6%)	1.24(0.93–1.65)
	>10 cars/min	578	34(5.9%)	1.64(1.11–2.41)	578	48(8.3%)	1.53(1.10–2.13)
							
NO_x _(μg/m^3^)	0–8	1855	74(4.0%)	1.00	1855	110(5.9%)	1.00
	8–11	1855	68(3.7%)	0.89(0.63–1.24)	1855	118(6.4%)	1.05(0.81–1.38)
	11–14	1855	87(4.7%)	1.19(0.86–1.64)	1855	121(6.5%)	1.12(0.86–1.46)
	14–19	1858	83(4.5%)	1.03(0.74–1.42)	1858	122(6.6%)	1.06(0.81–1.39)
	>19	1851	101(5.5%)	1.43(1.04–1.95)	1851	162(8.8%)	1.55(1.21–2.00)
							
			p-trend	0.010		p-trend	<0.0001

^a ^Adjusted for age, sex, and smoking. [OR(95%CI)].

**Table 6:** Geographical stratification. COPD diagnosis in Malmö and the region outside.

		Malmö			Region outside Malmö		
		n	COPD	OR ^a^	n	COPD	OR ^a^
Heavy traffic	No	1767	85(4.8%)	1.00	4178	152(3.6%)	1.00
	Yes	1877	103(5.5%)	1.24(0.92–1.67)	1343	69(5.1%)	1.47(1.09–1.97)
							
Heaviest road within <100 m	no heavy road	586	28(4.8%)	1.00	3124	124(4.0%)	1.00
	<2 cars/min	1021	44(4.3%)	0.89(0.55–146)	1193	49(4.1%)	1.06(0.75–1.49)
	2–5 cars/min	837	35(4.2%)	0.89(0.53–1.48)	961	35(3.6%)	0.93(0.64–1.37)
	6–10 cars/min	663	50(7.5%)	1.53(0.95–2.48)	212	10(4.7%)	1.20(0.62–2.35)
	>10 cars/min	537	31(5.8%)	1.34(0.79–2.28)	31	3	-
							
NO_x _(μg/m^3^)	0–8	13	0	-	1824	72(3.9%)	1.00
	8–11	46	2	-	1792	66(3.7%)	0.90(0.64–1.27)
	11–14	562	27(4.8%)	1.00	1268	60(4.7%)	1.26(0.89–1.80)
	14–19	1325	64(4.8%)	0.94(0.59–1.49)	510	18(3.5%)	0.91(0.54–1.55)
	>19	1698	95(5.6%)	1.23(0.79–1.92)	127	5(3.9%)	1.19(0.47–3.02)
							
			p-trend	0.142		p-trend	0.421

^a ^Adjusted for age, sex, and smoking. [OR (95%CI)].

**Table 7:** Geographical stratification. Chronic bronchitis symptoms in the city of Malmö and the area outside.

		Malmö			Region outside Malmö		
		n	Chronic b. symptoms	OR ^a^	n	Chronic b. symptoms	OR ^a^
Heavy traffic	No	1767	150(8.5%)	1.00	4178	246(5.9%)	1.00
	Yes	1877	140(7.5%)	0.91(0.71–1.16)	1343	92(6.9%)	1.20(0.94–1.54)
							
Heaviest road within <100 m	no heavy road	586	43(7.3%)	1.00	3124	179(5.7%)	1.00
	<2 cars/min	1021	89(8.7%)	1.21(0.83–1.77)	1193	68(5.7%)	1.00(0.75–1.34)
	2–5 cars/min	837	66(7.9%)	1.10(0.73–1.64)	961	69(7.2%)	1.30(0.98–1.74)
	6–10 cars/min	663	47(7.1%)	0.94(0.61–1.45)	212	19(9.0%)	1.63(0.99–2.69)
	>10 cars/min	537	45(8.4%)	1.22(0.78–1.89)	31	3	-
							
NO_x _(μg/m^3^)	0–8	13	0	-	1824	109(6.0%)	1.00
	8–11	46	4	-	1792	113(6.3%)	1.04(0.79–1.37)
	11–14	562	35(6.2%)	1.00	1268	84(6.6%)	1.17(0.87–1.57)
	14–19	1325	96(7.2%)	1.13(0.76–1.70)	510	26(5.1%)	0.88(0.57–1.37)
	>19	1698	155(9.1%)	1.57(1.06–2.30)	127	6(4.7%)	0.86(0.37–2.01)
							
			p-trend	0.017		p-trend	0.541

^a ^Adjusted for age, sex, and smoking. [OR(95%CI)].

### Association with traffic intensity on heaviest road within 100 m

Asthma diagnosis and asthma symptoms were associated with traffic intensity (table 2), with higher prevalence of asthma symptoms among those living next to a road with at least 6 cars/minute, and higher prevalence of asthma diagnosis among those exposed to at least 10 cars/minute, compared with the group having no road within 100 m. The effects seemed consistent, although statistically non-significant, across geographical region (tables 3, 4).

COPD and chronic bronchitis symptoms were associated with traffic intensity (table 5). However, when stratified geographically, the effect estimates indicated that chronic bronchitis symptoms were not associated with traffic intensity in Malmö (table 7).

### Association with NO_x _at residential address

Asthma symptoms, but not asthma diagnosis, were associated with NO_x _in the trend tests (table 2). However, effects were only seen in the highest quintile of >19 μg/m^3^. A geographical stratification showed that it was only in Malmö that high exposure was associated with asthma; no association was found in the region outside (tables 3, 4).

COPD diagnosis and chronic bronchitis symptoms were associated with NO_x_(table 5). After geographical stratification, associations were seen only in Malmö, and not in the region outside (tables 6, 7).

#### Stratification by smoking, sex, age, response group, and restricted analysis

In a stratified analysis, the effects of traffic exposure were more pronounced for smokers than for non-smokers, for both COPD (table 8) and bronchitis symptoms (data not shown). A test for interaction, however, showed no significance except for the interaction between smoking and road within 100 m for chronic bronchitis symptoms (p = 0.023). Asthma showed no indications of effect modification by smoking.

**Table 8:** Stratification on smoking. COPD diagnosis in relation to traffic among smokers/ex-smokers and non-smokers.

		Smokers/ex-smokers			Non-smokers		
							
		n	COPD D	OR ^a^	n	COPD D	OR ^a^
Heavy traffic	No	3149	167(5.3%)	1.00	2892	76(2.6%)	1.00
	Yes	1861	130(7.0%)	1.43(1.13–1.82)	1414	42(3.0%)	1.19(0.81–1.76)
							
Heaviest road within <100 m	no heavy road	1951	104(5.3%)	1.00	1804	49(2.7%)	1.00
	<2 cars/min	1185	67(5.7%)	1.06(0.77–1.45)	1050	28(2.7%)	0.99(0.62–1.59)
	2–5 cars/min	992	52(5.2%)	0.99(0.70–1.40)	828	19(2.3%)	0.88(0.51–1.51)
	6–10 cars/min	522	44(8.4%)	1.56(1.08–2.26)	364	16(4.4%)	1.64(0.92–2.94)
	>10 cars/min	344	28(8.1%)	1.84(1.18–2.86)	234	6(2.6%)	1.15(0.48–2.75)
							
NO_x _(μg/m^3^)	0–8	969	47(4.9%)	1.00	886	27(3.0%)	1.00
	8–11	971	47(4.8%)	0.96(0.63–1.46)	884	21(2.4%)	0.77(0.43–1.37)
	11–14	945	63(6.7%)	1.35(0.92–2.00)	910	24(2.6%)	0.92(0.52–1.61)
	14–19	1037	60(5.8%)	1.14(0.92–2.00)	821	23(2.8%)	0.85(0.48–1.50)
	>19	1072	78(7.3%)	1.61(1.11–2.35)	779	23(3.0%)	1.12(0.63–1.98)
							
Test för	Heavy traffic*eversmoker			p = 0.47			
Interaction	Heaviestroad100 m*eversmoker			p = 0.89			
	NOx*eversmoker			p = 0.83			

^a ^Adjusted for age and sex. [OR(95%CI)].

No effect modifications were seen when the data were stratified by sex, age, or sample group (new participants vs. follow-up group). Restriction of analysis to asthmatics without COPD, and to those with COPD without asthma, was performed for both diagnoses and symptoms. The results showed similar effects in the restricted and non-restricted groups. The overlaps between the different disease outcome definitions are presented in table 9.

**Table 9:** Description of overlap between the different reported disease outcomes. Percentage within row. The first row shows that 70% of those with asthma diagnosis had asthma symptoms, 20% of those with asthma diagnosis had COPD diagnosis, and 21% of those with asthma diagnosis had chronic bronchitis symptoms.

	Total n	Asthma diagnosis n (%)	Asthma symptoms n (%)	COPD diagnosis n (%)	Chronic b. Symptoms (n %)
Asthma diagnosis	686	-	483 (70%)	139 (20%)	145 (21%)
Asthma symptoms	1115	483 (43%)	-	219 (20%)	277 (25%)
COPD diagnosis	415	139 (34%)	219 (53%)	-	152 (37%)
Chronic bronchitis symptoms	635	145 (23%)	277 (44%)	152 (24%)	-

## Discussion

Overall, residential traffic was associated with a higher prevalence of both asthma diagnosis and asthma symptoms in the last 12 months, as well as COPD diagnosis and chronic bronchitis symptoms. Traffic intensity on the heaviest road within 100 m showed effects at a traffic intensity of >6 cars/min. Effects from NO_x _were seen in the highest exposure quintile of >19 μg/m^3^, but only in Malmö, not in the region outside.

### Discussion of exposure assessment

The major strength of this study was the use of three different proxies of exposure, which may have different intrinsic strengths and weaknesses. The strengths of the NO_x _model are firstly that it reflects total traffic density in the area, and secondly the fact that the dispersion model has been validated, with a resolution of 250 × 250 m showing a high correlation with measured background concentrations [25]. Nevertheless, street-level concentrations may vary on a much smaller scale. High peak concentrations are often found in so-called "street canyons" in urban areas, where pollutants are trapped between high buildings [23]. Since the dispersion model did not take account of this kind of accumulation effect, the true exposure among people living in these surroundings might have been underestimated. This may partly explain why effects from NO_x _were seen in the urban city of Malmö but not in the surrounding area.

The proportion of NO_x _that originates from traffic is also dependent on geographical area. In urban areas of southern Sweden, local traffic contributes approximately 50–60% of total NO_x_, while in the countryside such traffic is responsible for only 10–30% of total NO_x _(S. Gustafsson, personal communication, 2007-10-17). This difference was also reported by the SAPALDIA study, which found that local traffic accounted for the majority of NO_x _in urban but not rural areas [35]. This indicates that our model of NO_x _is a good proxy for exposure to traffic-related air pollution in an urban area, but may not be sensitive enough to capture individual risk in the countryside, where traffic contributes to a lower proportion of total concentrations.

Self-reported living close to a road with heavy traffic, and traffic intensity on the heaviest road within 100 m, are simple proxies; they do not reflect, for example, whether someone lives at a junction. Still, they have the advantage that they are less limited by aggregation in space than the NO_x _model. In the present study, both of these variables showed fairly consistent associations, at least with asthma, despite large differences in the level of NO_x _that they corresponded to in Malmö and the region outside (table 10); this may indicate that adverse effects from traffic pollutants are mainly seen in close proximity to traffic. High traffic intensity, however, may not only correlate with high total number of vehicles, but also with a higher proportion of heavy vehicles, an additional factor which could affect the outcome, since diesel exhaust from heavy vehicles might have more adverse respiratory effects [36].

**Table 10:** Relation between the exposure proxies and modeled NO_x _(μg/m^3^) as a continuous variable.

		Malmö NO_x_				Region outside Malmö NO_x_			
		n	Mean	SD	Median	n	Mean	SD	Median
									
Heavy traffic	No	1507	18.0	3.1	17.4	4502	10.2	3.5	9.6
	Yes	1772	19.6	3.2	19.6	1495	12.1	4.5	11.4
									
Heaviest road within <100 m	no heavy road	488	17.6	3.4	17.2	3267	10.1	3.4	9.6
	<2 cars/min	855	18.0	2.9	17.8	1380	9.8	4.3	8.1
	2–5 cars/min	746	18.9	3.3	19.4	1074	12.6	3.8	11.5
	6–10 cars/min	627	18.1	2.8	17.4	259	13.8	2.3	14.03
	>10 cars/min	561	21.9	2.0	22.0	17	19.2	4.4	21.6
									
NO_x _(μg/m^3^)	0–8	13	6.8	1.3	6.8	1824	6.7	1.1	6.8
	8–11	46	10.4	0.8	9.6	1792	9.9	0.8	10.0
	11–14	562	13.5	0.7	13.7	1268	12.8	1.0	12.7
	14–19	1325	16.7	1.3	15.9	510	15.7	1.2	15.3
	>19	1698	21.7	1.3	21.5	127	21.9	3.8	21.2
									
	Total	3644	18.4	3.6	18.5	5521	10.31	3.6	10.04

It should be noted that the distribution of exposure is not comparable between the proxies. While NO_x _was divided into quintiles, with 20% in the highest exposure category, only 6% of the population lay in the highest traffic intensity category. Thus, the different proxies are complementary rather than comparable in this study.

One limitation of all three proxies of exposure was that traffic-related air pollution was only estimated by residential address. Lack of individual data about work address and time spent commuting could have biased the exposure assessments, particularly for people living in areas with low exposure to traffic-related air pollution, where individual differences in daily activities outside the residential area translate to a large proportion of total exposure [37]. In Los Angeles, 1 h commuting/day contributes approximately 10–50% of people's daily exposure to ultrafine particles from traffic [38]. While only 20% of the working population living in Malmö commute to work outside Malmö, the majority of the population in smaller municipalities in the remaining region commute to work outside their own municipality [39].

Another limitation was the cross-sectional nature of the study; we had no information about disease onset or years living at current address, making it hard to establish a temporal relationship between cause and effect. However, since asthma and COPD are known to be exacerbated by traffic-related air pollution, subjects with disease may have been more likely to move away from traffic, rather than towards it, and so a migrational bias would mainly be expected to dilute the effects.

### Discussion of potential confounding

Areas with high levels of exposure to traffic-related air pollution were mainly located in the city of Malmö (table 4 and figure 2), while low exposure was found in more sparsely populated areas. It is a well recognized problem that the different exposure levels in rural and urban environments are also accompanied by large differences in lifestyle factors, and even if confounders are controlled for, unmeasured factors may remain. Since NO_x _was limited by its spatial resolution, it is also the measure that was most susceptible to ecological bias. The lack of association seen with NO_x_, in the region outside Malmö might thus reflect that the individual risk from traffic is being overridden by some other factor correlating with low exposure levels. The existence of independent risk factors correlating with low exposure is given some support by a Swedish study which found a tendency to higher adult asthma incidence in rural areas, after adjustment for exposure to traffic [11].

The most important risk factors from a validity standpoint, however, are factors that could correlate with high exposure to traffic-related air pollution, and thus cause a false positive relationship, such as socio-economic and occupational risk factors. However, the present study, which used individual-level data, found no confounding effects for either socio-economic status or occupational exposure. A recently developed and validated JEM was used to adjust for occupational exposure [33]. In a JEM, people are assigned the statistically average level of exposure in their occupation; this is an aggregated form of exposure assessment that can suffer from misclassification bias, although non-differential to disease. Since we only had information on the participants' current occupations, we cannot rule out the possibility of a "healthy worker effect". Lack of information about occupational history may be a limitation, especially in relation to the prevalence of COPD/chronic bronchitis.

## Results discussion

Although asthma and COPD have many risk factors in common and often coexist in clinical settings, and there is some evidence that asthma may be a risk factor for the development of COPD [40], they are distinct conditions, with differing clinical course and pathological features. Thus, inconsistencies between studies in the relation between air pollution and asthma/COPD could depend both on the presence of different competing risk factors, and on geographically different pollution mixtures acting on different regions of the respiratory tract. It is therefore important to consider local pollution characteristics as thoroughly as possible (tables 11, 12). When using traffic intensity or self-reported traffic exposure as a proxy, there is a lack of knowledge of the exact pollution compounds that this exposure represents. One known characteristic of traffic-related pollution in the study region is a large amount of wear particles from road-tire interaction. These particles have been shown to be potent inducers of local inflammation [41,42], and their levels are high in the Scandinavian countries due to the use of traction sand and studded tires.

**Table 11:** Urban background. Descriptive data of regional air pollution at a monitoring station in Malmö. Annual mean concentrations of traffic-related pollutants measured at Rådhuset Malmö 1980–2006. Data source: IVL Swedish Environmental Research Institute Ltd.

Year	SO_2 _(μg/m^3^)	NO_2 _(μg/m^3^)	O_3 _(μg/m^3^)	PM_10 _(μg/m^3^)	PM_2.5 _(μg/m^3^)
1980*	49				
1981	50				
1982	43				
1983	33,1				
1984	22,9	42			
1985	20,3	39			
1986	16,7	31			
1987	20,3	32			
1988	13	30.5			
1989	12	26.9	46		
1990	9	21.3	39		
1991	8	19.6	41		
1992	7	22.4	43		
1993	8	25.6	40		
1994	6	21.4	43		
1995	6	22	50		
1996	8	24.6	50	17.4	
1997	5	26.2	48	17.6	
1998	4	21.8	47	15.2	
1999	4	23.5	50	15.8	12.6
2000	2	22.9	49	16.5	13.5
2001	2	21.1	46	18.7	12
2002	2	20.3	52	18.1	11.5
2003	3	20.8	49	21.6	13.7
2004	3	19.5	54	15.9	10
2005	4	20.6	49	17.5	11.1
2006	3	19.3	52	18.2	12.3

**Table 12:** Rural background. Descriptive data of regional air pollution at a monitoring station in a rural area. Annual mean concentrations of traffic-related pollutants measured at Vavihill 1985–2006. Data source: IVL Swedish Environmental Research Institute Ltd.

Year	SO_2 _(μg/m^3^)	NO_2 _(μg/m^3^)	O_3' _(μg/m^3^)	PM_10 _(μg/m^3^)	PM_2.5 _(μg/m^3^)
1985	5.14	2.36	60.2		
1986		2.27	59.9		
1987	5.47	2.11	55.1		
1988	3.90	1.84	57.7		
1989	3.93	2.66	56.5		
1990	2.98	2.36	55.0		
1991	2.64	2.08	51.3		
1992	2.06	1.72	56.0		
1993	1.70	1.98	57.4		
1994	1.17	1.78	58.6		
1995	1.35	1.92	59.3		
1996	1.31	1.77	63.0		
1997	0.67	2.05	58.8		
1998	0.74	1.87	54.6		
1999	0.55	1.66	59.1		
2000	0.45	1.70	57.6	16.0	
2001	0.42	1.37	60.2	15.4	
2002	0.37	1.39	66.6	16.3	
2003	0.52	1.54	62.9	18.6	
2004	0.37	1.48	58.5	13.8	
2005	0.49	1.47	61.0	15.2	
2006	0.50	1.59	64.3	17.3	

Although levels of traffic pollution in Sweden are lower than those found in most other countries, the results for asthma are basically supported by some European studies with higher exposure levels. An Italian study reported an association between symptom exaggeration of adult asthma and NO_2 _exposure levels [12], and the Swiss SAPALDIA study observed an increase of asthma-related symptoms, although not current asthma, in relation to NO_2 _[43]. The European ECRHS study found a positive association between NO_2 _(modeled with a resolution of 1 km) and asthma incidence, but effect estimates seemed very heterogenous among the Swedish centers (although overall heterogeneity tested was non-significant). [15]. Most relevantly, a Swedish study found a non-significant tendency to increased asthma incidence among adults living close to traffic flows, and measured NO_2 _levels comparable to those found in the present study [11]. Another study of asthma symptoms in Sweden found a significant but weak relation to NO_2 _[44], although a stronger relation was found with self-reported measures of traffic. The findings in the present study, support the existence of a relation between exposure to traffic-related air pollution and asthma in adults at relatively low levels of traffic-related air pollution.

For COPD, a German study restricted to women found that COPD as defined by the GOLD criteria was 1.79 times more likely (95% CI 1.06–3.02) for those living less than 100 m from a road with 10 000 cars/day, than for those living farther away [19]. This is in agreement with our results, which found effects for living less than 100 m from a road with 6 cars/min (8 640 cars/day).

The European ECRHS study found that new onset of chronic bronchitis, as defined by chronic phlegm, was related among females to both self-reported traffic intensity (constant traffic vs. none, OR = 1.86; 95% CI 1.24 to 2.77) and home outdoor NO_2 _(OR = 50 μg/m^3 ^vs. 20 μg/m^3 ^= 2.71; 95% CI 1.03 to 7.16) [20]. The higher levels of NO_2 _seen in the ECRHS study may partly stem from truly higher concentrations, but may also have been affected by the use of home outdoor measurements, which are better than our models at capturing locally high peak exposures. Other studies have suggested an effect modification for sex, with women being at higher risk, but this was not observed in our study. Our results did indicate effect modification by smoking, but tests for interaction were mainly non-significant. No interaction with smoking was found in any of the abovementioned studies of the effects of air pollution on prevalence/incidence of COPD in adults.

Overall, our results show that traffic-related air pollution is associated with the prevalence of COPD/chronic bronchitis in adults, but there is still a need for further investigation of the reasons behind the inconsistencies seen when the data were stratified by region.

## Conclusion

Residential traffic is associated with both current symptoms and prevalence of diagnosis of asthma and COPD/chronic bronchitis, among adults in southern Sweden. This may indicate that traffic has not only short-term but also long-term effects on adult chronic respiratory disease, even in a region with low overall levels of traffic pollution.

## Competing interests

The authors declare that they have no competing interests.

## Authors' contributions

AL: Conducted the statistical analyses and wrote the main part of the manuscript. ES: Performed GIS analyses and wrote part of the manuscript. PM: Designed and conducted the survey and made revisions on drafts. UN: Designed and conducted the survey and made revisions on drafts. KJ: Designed the study and made revisions on drafts. AA: Wrote part of the manuscript and made major revisions of drafts. All authors read and approved the final manuscript.
